# Intracranial hemorrhages and late hemorrhagic disease associated cholestatic liver disease

**DOI:** 10.1007/s10072-012-0965-5

**Published:** 2012-02-11

**Authors:** Hüseyin Per, Duran Arslan, Hakan Gümüş, Abdulhakim Çoskun, Sefer Kumandaş

**Affiliations:** 1 Department of Pediatric Neurology, Medical Faculty, Erciyes University, Erciyesevler Mahallesi, 30 Ağustos Caddesi, Köknar Sokak, Saklıbahçe Sitesi, No 3/19, Kayseri, Turkey; 2 Department of Pediatric Gastroenterology, Medical Faculty, Erciyes University, Kayseri, Turkey; 4 Department of Pediatric Neurology, Medical Faculty, Erciyes University, Kayseri, Turkey; 3 Department of Pediatric Radiology, Medical Faculty, Erciyes University, Kayseri, Turkey

**Keywords:** Intracranial bleeding, Late hemorrhagic disease, Liver disease

## Abstract

Deficiency of vitamin K predisposes to early, classic or late hemorrhagic disease of the newborn (HDN); of which late HDN may be associated with serious and life-threatening intracranial hemorrhage. Late HDN is characterized intracranial bleeding in infants aged 1 week to 6 months due to severe vitamin K deficiency. Late HDN is still an important cause of mortality and morbidity in developing countries where vitamin K prophylaxis is not routinely practiced. Children with cholestatic liver disease are at risk for developing secondary vitamin K deficiency because of fat malabsorbtion and inadequate dietary intake. In this study, we described 11 infants with cholestatic liver disease with different etiologies exhibiting intracranial hemorrhage (ICH). Six patients underwent surgical evacuation of ICH, following the administration of vitamin K and/or fresh frozen plasma. The possibility of cholestatic liver disease should be considered in the treatment of ICH due to vitamin K deficiency.

## Introduction

Vitamin K is a fat-soluble vitamin that is necessary for the synthesis of factors II, VII, IX, X in the liver. Vitamin K can pass through the placenta very poorly and is present only in very low concentrations in human milk. Breast-fed infants do not receive the recommended vitamin K intake via human milk. In addition, the intestinal flora of the breast-fed infants may produce less vitamin K than the flora of the formula-fed infants [1–3].

Hemorrhagic disease of the newborn (HDN) caused by vitamin K deficiency has been recognized as an important cause of morbidity and mortality. HDN is one of the most common causes of acquired hemostatic disorder in early infancy. HDN in infancy comprises early (0–24 h), classical (1–7 days), and late (1 week to 6 months) syndromes according to the time of presentation. Late HDN is characterized by bleeding in infants aged 1 week to 6 months due to severe vitamin K deficiency but can occur anytime in the first year, occurring primarily in exclusively breast-fed infants [4–8]. Known risk factors include breastfeeding and the failure to give vitamin K prophylaxis at birth. The association between late HDN and abnormalities of liver function has also been reported in surveillance programmes from several countries [4, 5, 9–15]. Several studies have indicated that certain cholestatic liver diseases such as biliary atresia (BA), neonatal hepatitis, α_1_ antitrypsin deficiency, cystic fibrosis may be responsible for the great majority of cases of late HDN [10, 15–19].

## Patients and methods

Between June 1995 and June 2005, 120 infants with late HDN were treated and followed up at Erciyes University Medical Faculty. One hundred and six of these patients presented with intracranial hemorrhage. Eleven of these 106 patients (10.3%) had apparent liver disease. Medical records of children with late HDN and liver disease were evaluated retrospectively.

Late HDN in a neonate defined as following: no thrombocytopenia, normal peripheral blood smear examination, prolonged prothrombin time (PT), and activated partial thromboblastin time (aPTT), which normalized within 12-24 h after administering vitamin K, was considered to have late HDN. Laboratory studies included a complete blood count, urine analysis, liver function test (AST, ALT, total and direct bilirubin, alkaline phosphatase, gamma glutamyl transpeptidase, protein and albumin levels), abdominal USG, cranial computerized tomography. For the differential diagnosis of liver disease blood and urine amino acids, urinary reducing substance, TORCH serology, sweet chloride test, α_1_ antitrypsin level, thyroid function tests, blood ammonia, private and lactate levels, hepatobiliary scintigraphy were done. Percutaneous liver biopsy was obtained either as surviving or postmortem.

**Table 1:** Some of the demographic, clinical and laboratory features of the patients

Patient no	Age (w)/sex	Presenting complaints	Feeding type	Bleeding site	PT aPTT	Laboratory^a^	Diagnosis/outcome
1	12/M	Epistaxis, bleeding injection area, seizure	Breastfeeding	Intracerebral hemorrhage	PT > 1 min aPTT > 1 min	Hb: 7.6 AST: 136, ALT: 148 D. Bil: 6.9	CMV hepatitis Died at 3 months of age
2	14/M	Bleeding injection area, seizure	Breastfeeding	Frontal hemorrhage	PT > 1 min PTT > 1 min	Hb: 8.1 AST: 97, ALT: 101 D. Bil: 7.7	CMV hepatitis Surviving without sequel
3	7/M	Vomiting, pitozis, seizure, irritability	Breastfeeding	Temporal hematoma	PT > 1 min PTT > 1 min	Hb: 5.6 AST: 203, ALT: 77 D. Bil: 4.9	No surgery Died at hospital follow-up
4	7/M	Vomiting, seizure, diarrhea	Breastfeeding	Subdural and intracerebral hematoma	PT > 1 min PTT > 1 min	Hb: 5.2 AST: 221, ALT: 312 D. Bil: 6.4	Giant cell hepatitis Operated for ICH, surviving, epilepsy, mental retardation
5	6/M	Jaundice, seizure	Breastfeeding	Subdural and intracerebral hematoma	PT > 1 min PTT > 1 min	Hb: 4.0 AST: 360, ALT: 250 D. Bil: 8.1	Neonatal hepatitis, operated for ICH Surviving, epilepsy, mental retardation
6	7/F	Vomiting, jaundice, seizure	Breastfeeding	Subdural hematoma	PT > 1 min PTT > 1 min	Hb: 6.5 AST: 250, ALT: 93 D. Bil: 9.2	Operated for ICH and BA, died from liver failure at 8 months of life from liver failure
7 Case 1	16/F	Jaundice, diarrhea, vomiting, seizure	Breastfeeding	Intracerebral hemorrhage	PT > 1 min PTT > 1 min	Hb: 7.8 ALT: 240, AST: 128 D. Bil: 10.6	Giant cell hepatitis No surgery Died at hospital follow-up
8	4/M	Poor feeding, irritability, vomiting, seizure	Breastfeeding	Subdural hematoma	PT > 1 min PTT > 1 min	Hb: 10.4 AST: 115, ALT: 86 D. Bil: 13.2	Operated for ICH and BA, died at follow-up
9	8/F	Vomiting, fever, seizure, bleeding injection area	Breastfeeding	Intracerebral hemorrhage	PT > 1 min PTT > 1 min	Hb: 7.3 AST: 178, ALT: 307 D. Bil: 12.7	Giant cell hepatitis, died at follow-up from liver failure
10 Case 2	4/F	Jaundice, pitozis, poor feeding, seizure	Breastfeeding	Subdural hematoma	PT > 1 min PTT > 1 min	Hb: 7.5 AST: 231, ALT: 171 D Bil. 12.9	Operated for ICH and BA, died from liver failure at 12 months of life from liver failure
11	3/M	Jaundice, bleeding injection area, seizure	Breastfeeding	Intracerebral and Intraventricular hemorrhage	PT > 1 min PTT > 1 min	Hb: 6.3 AST: 239, ALT: 420 D. Bil: 9.9	Operated for ICH and BA, died at follow-up

^a^Hemoglobin, g/dl; AST and ALT, IU/L; direct bilirubin, mg/dl; median (and minimum–maximum) values for age, hemoglobin, AST, ALT and direct bilirubin levels are 7 (3–16) weeks, 7.3 (4.0–10.4) g/dl, 221 (97–360) IU/L, 148 (77–420) IU/L and 9.2 (4.9–13.2) mg/dl, respectively

A 3 mg of vitamin K was administered by intravenous route to all patients on admission. Fresh frozen plasma was also transfused especially to preoperative period as required. PT was normalized after 24 h of administration of vitamin K in all patients, and no additional bleeding was observed. Seven of the patients were male and 4 of them were female. The ages of the patients were between 3 and 16 weeks (median 7 week). All patients were having breastfeeding. Final diagnoses of infants were BA in four, cytomegalovirus hepatitis in two, giant cell hepatitis in three and neonatal hepatitis in two. Six of the patients were suitable for surgery and operated for intracranial hemorrhage (ICH), and then four patients with the final diagnosis of BA had Kasai procedure. Properties of the patients have been shown in Table 1.

### Case 1

A 58-day-old female with 8-hour history of irritability, fever, diarrhea, seizure, jaundice, and intensive vomiting was admitted. She had no complaint for the previous days. She was born at term with a normal vaginal birth with the help of a midwife at home. After uncomplicated pregnancy, exclusively breast-feed, thriving, the infant was on no medication. Routine vitamin K was not administered at birth. She was not yet old enough to have started primary immunization. She had no history of bleeding. There was no kinship between mother and father. The mother had no medication during the pregnancy. There was no hemorrhagic diathesis disorder in his family.

She had fever (38.4°C), irritable, tachycardic (148/min), tachypneic (54/min). Her blood pressure was 70/30 mm Hg. She was uncomfortable and pale and jaundiced. On admission to hospital on physical examination, she revealed tense and bulging anterior fontanels, bleeding from injection area, hepatomegaly, anisocoria and increased deep tendon reflexes.

**Fig. 1:**
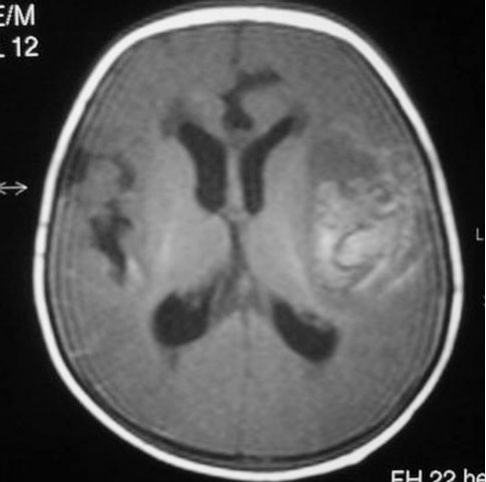
Axial T_1_-weighted cranial MR image shows hemorrhages in temporal lobe

The results of the laboratory studies are as follows: white blood cell count 16,400/mm^3^ with 70% neuthrophils and 30% lymphocytes. Hemoglobin 7.8 g/dl; platelet count 408,000/mm^3^; direct bilirubin 10.6 mg/dl; aspartate aminotransferase 240 IU/L; alanine aminotransferase 128 IU/L; both the activated partial thromboplastin time and the prothrombin time are longer than 1 min and seronegative for human immunodeficiency virus, cytomegalovirus and Epstein Barr virus. Twelve hours later following the administration of 3 mg vitamin K, activated partial thromboplastin time was detected to be 33 s; prothrombin time was detected to be 14 s. Cranial MR images demonstrated intracerebral hemorrhage in the left temporal lobe (Fig. 1).

Intravenous vitamin K and erythrocyte suspension was added to treatment. She died 44 h after following her admission to the hospital. Postmortem liver biopsy was demonstrated giant cell hepatitis.

### Case 2

A 29-day-old female was brought to pediatric emergency unit with the complaints of poor feeding, jaundice, vomiting, seizure, pitozis and prolonged bleeding from the injection site. She was born at home with the help of a midwife. She had no history of diarrhea or drug use. The baby was exclusively breast-fed. Her mother did not use any medication during the pregnancy period.

She had no fever (36.8°C), irritable, tachycardic (154/min), tachypneic (47/min). Her blood pressure was 90/50 mm Hg. She was uncomfortable and pale. On admission to our hospital on physical examination, she revealed a tense and bulging anterior fontanels, isochoric pupils, hepatomegaly, increased deep tendon reflexes. No bruises, petechia or ecchymosis were observed, except in intramuscular injection site.

Laboratory investigations were as follows: white blood cell count 13,800/mm^3^; hemoglobin 7.5 g/dl; platelet count 551,000/mm^3^; aspartate aminotransferase 231 IU/L; alanine aminotransferase 171 IU/L; alkaline phosphatase 474 U/L; direct bilirubin 9.9 mg/dl; both activated partial thromboplastin time and prothrombin time are longer than 1 min. The patient was seronegative for human immundeficiency virus, hepatitis B, cytomegalovirus and Epstein-Barr virus. Twelve hours later following the administration of 3 mg vitamin K, activated partial thromboplastin time was 32 s and prothrombin time was 11 s. Urine analysis and blood urea nitrogen and creatinine were normal. The results of renal function and urine analysis were in the normal range. Cranial CT scan demonstrated subdural hemorrhage. Erythrocyte suspension, anti-edema and anti-anticonvulsant treatments were given and 3 mg of vitamin K was administered intravenously. Eighteen hours after the admission, PT was 11 s and aPTT was 26 s. Hepatobiliary scintigraphy revealed no radioactivity passed to intestine. Percutaneous liver biopsy showed intracellular and intracanalicular bile thrombi, bile duct proliferation and widening of portal space consistent with BA. She had Kasai procedure at 2 months of age. She died at 12 months of age from liver failure.

## Discussion

Late HDN, an important cause of morbidity and mortality, manifests after the second week of life and is particularly associated with ICH. The rate of late HDN ranges from 4.4 to 72 cases per 100,000 births (not given vitamin K prophylaxis) based on reports from Europe and Asia. Incidence of late HDN in the eastern world is 25–80/100,000 births, which is higher than that in the western world (4–25/100,000 births) [20]. In developed countries HDN is now a rare life-threatening disease due to the widespread use of effective prophylaxis with vitamin K at birth. The postnatal administration of vitamin K has dramatically reduced the incidence of vitamin K deficiency bleeding during the first weeks of life; although sporadic cases with late onset hemorrhage are described among exclusively breast-feed infants who did not receive additional prophylaxis. Routine vitamin K prophylaxis at birth brought down the incidence of late HDN from 7/100,000 to 1.1/100,000 live births in Netherlands [11, 12, 21].

Late HDN may be primary or secondary to cystic fibrosis, BA, α_1_ antitrypsin deficiency, hepatitis, abetalipoproteinemia, celiac disease or chronic warfarin exposure [1–4, 7, 12, 22, 23]. Children with cholestatic liver disease are at risk for developing vitamin K deficiency because of fat malabsorption and inadequate dietary intake. In secondary late HDN additional factors can be demonstrated, such as poor intake or absorption of vitamin K and increased consumption of vitamin K [17, 24].

Although idiopathic vitamin K deficiency has decreased markedly by introduction of prophylactic vitamin K administration [3, 4], prophylaxis has no effect on secondary vitamin K deficiency caused by malabsorption of vitamin K in the digestive tract. The incidence of secondary vitamin K deficiency has not decreased [5]. The true prevalence of vitamin K deficiency in cholestatic liver disease is not known and recommendations regarding routine supplementation are therefore controversial. Vitamin K deficiency is prevalent in children with mild-to-moderate cholestatic liver disease, even with vitamin K supplementation [17]. Vitamin K deficiency is related to degree of cholestasis and severity of liver disease in children [17]. Adequate vitamin K status in children with cholestatic liver disease is critical to support coagulation and probably brain development. Vitamin K deficiency places the child with cholestatic liver disease at risk for life-threatening hemorrhage which can lead to disability and death [17, 24].

The causes of secondary vitamin K deficiency include chronic diarrhea, long-term antibiotic therapy, and hepatobiliary lesions such as BA and neonatal hepatitis. Cholestasis was detected in 37% of the 108 with HDN patients in a report from Germany by Sutor et al. [5]. Ekelund [10] reported 17 cases with late HDN in a population of 332.686 Swedish babies. Fifteen had cholestatic liver disease. There were three cases of α_1_ antitrypsin deficiency, five BA, and seven with other conditions. Loughnan and Dougall [13] reported cholestatic liver disease in 55 of 131 children with late HDN. There were 22 cases of α_1_ antitrypsin deficiency, 11 BA, and 22 other liver diseases. In our study, we described 11 infants (4 female, 7 male) with cholestatic liver disease with different etiologies presenting with ICH. Final diagnoses of our patients were BA in four, giant cell hepatitis in three and neonatal hepatitis in two and cytomegalovirus hepatitis in two.

Although it has been reported that BA is one of the major causes of secondary vitamin K deficiency [25], there are few reports of patients with BA presenting with ICH [14, 18, 25, 26]. Akiyama et al. [18] described 15 patients of BA presenting with ICH. They suggest that surgical intervention for ICH has no adverse effects on subsequent surgical management of BA. Sato et al. [25] reported a 2-month-old female suffering from ICH due to secondary vitamin K deficiency associated with BA, who had undergone craniotomy successfully 1 h after administration of both vitamin K and frozen human plasma. The patients with BA constitute the biggest group of our patients (36%). All of them had successful Kasai procedure but at follow-up they did not have liver transplantation. Unfortunately they died from liver failure in 2 years after birth. In the long-term follow-up, it was reported that some neurological sequel such as developmental delay, mental retardation, and epilepsy was observed in more than half of patients with ICH due to vitamin K deficiency. In Akiyama’s series, neurological sequel including mental retardation and epilepsy remained in 2 of 15 patients, while 3 patients were noted to have hemiparesis in the early stage they recovered completely during follow-up [18].

Intracranial hemorrhage can be presented as the manifestation of late HDN in children leading to death in some cases and neurological sequel in the survivors. Almost 2/3rd of the infants with late HDN present with serious ICH leading to high morbidity and subsequent mortality. In a study performed in Turkey, neurological findings were found in 73% of the patients, mortality was 33% [27]. Another study including 14 cases in India showed that 88% of cases had ICH and mortality rate was 57% [28]. Also in another review of 108 cases of late HDN from Germany, 58% had ICH with a mortality of 19% and neurological sequel in 21% [5]. A prospective study, conducted for over 2 years in the British Isles, found that 10 out of the 27 infants had ICH, and 2 of these with intracranial bleed died [15]. We lost six of our cases in our follow-up, two of them did not come to control, and motor retardation and epilepsy are being followed in three of them.

Since late HDN leads to significant morbidity and mortality, providing vitamin K prophylaxis to all newborns should prevent it. Administration of vitamin K (1 mg) at birth can inhibit intracranial bleeding and other hemorrhagic manifestations [29]. The recommendation of the American Academy of Pediatrics is to give vitamin K to all newborns as a single intramuscular dose of 0.5–1.0 mg [30]. The standard manner of vitamin K administration in Turkey is 1 mg intramuscular vitamin K administration at birth. When vitamin K deficiency is suspected, vitamin K should be administered immediately, with rapid correction of PT and aPTT confirming the diagnosis. It has been reported that intravenous administration of vitamin K 0.5–1.0 mg/kg improves clinical bleeding tendency within 1 h and normalizes coagulation within several hours [1, 6].

In conclusion, children with late HDN should be evaluated for cholestatic liver disease including biliary atresia. Vitamin K deficiency may increase the risk for life-threatening hemorrhage in children with cholestatic liver disease, which can lead to disability and death. Therefore, vitamin K supplementation is crucially important in patients with cholestatic liver disease.

## References

[1] Shearer MJ (1992). Vitamin K. Metabolism and nutriture. Blood Rev.

[2] Lane PA, Hathaway WE (1985). Vitamin K in infancy. J Pediatr.

[3] Greer FR (1999). Vitamin K status of lactating mothers and their infants. Acta Paediatr Scan Supply.

[4] Sutor AH, Von Kries R, Cornelissen EAM, McNinch AW, Andrew M (1999). Vitamin K deficiency bleeding (VKDB) in infancy. Thromb Haemost.

[5] Sutor AH, Dagres N, Neiderhoff H (1995). Late form of vitamin K deficiency bleeding in Germany. Klin Pediatr.

[6] Sutor AH (1995). Vitamin K deficiency bleeding in infants and children. Semin Thromb Hemost.

[7] Greer FR (1995). Vitamin K deficiency and hemorrhage in infancy. Clin Perinatol.

[8] Per H, Kumandas S, Ozdemir MA, Gumus H, Karakukcu M (2006). Intracranial hemorrhage due to late hemorrhagic disease in two siblings. J Emerg Med.

[9] Hanawa Y, Maki M, Murata B, Matsuyama E, Yamamoto Y, Nagao T (1988). The second nation-wide survey in Japan of vitamin K deficiency in infancy. Eur J Pediatr.

[10] Ekelund H (1991). Late haemorrhagic disease in Sweden 1987–89. Acta Pediatr Scand.

[11] Cornelissen EAM, Hirasing RA, Monnens LA (1996). Prevalence of hemorrhages due to vitamin K deficiency in Netherlands 1992–1994. Ned Tijdschr Geneeskd.

[12] Lulseged S (1993). Haemorrhagic disease of the newborn: a review of 127 cases. Ann Trop Paediatr.

[13] Loughnan PM, Mc Dougall PN (1993). Epidemiology of late onset haemorrhagic disease: a pooled data analysis. J Paediatr Child Health.

[14] Matsuzaka T, Yoshinaga M, Tsuji Y, Yasunaga A, Mori K (1989). Incidence and causes of intracranial hemorrhage in infancy: a prospective surveillance study after vitamin K prophylaxis. Brain Dev.

[15] McNinch AW, Tripp JH (1991). Haemorrhagic disease of the newborn in the British Isles: two year prospective study. BMJ.

[16] Schubiger G, Stacker C, Banziger O, Laubscher B, Zimmermann H (1999). Oral vitamin K1 prophylaxis for newborns with a new mixed-micellar preparation of phylloquinone: 3 years experience in Switzerland. Eur J Pediatr.

[17] Mager D, McGee PL, Furuya KN, Roberts EA (2006). Prevalence of vitamin K deficiency in children with mild to moderate chronic liver disease. J Pediatr Gastroenterol Nutr.

[18] Akiyama H, Okamura Y, Nagashima T, Yokoi A, Muraji T, Uetani Y (2006). Intracranial hemorrhage and vitamin K deficiency associated with biliary atresia: report of 15 cases and review of the literature. Pediatr Neurosurg.

[19] Auerswald G, Sutor AH, Sutor AH, Hathaway WE (1995). Thirteen cases of α1-antitrypsin deficiency presenting as a bleeding diathesis with intracranial hemorrhage in the newborn. Vitamin K infancy.

[20] von Kries R, Hanawa Y (1993). Neonatal vitamin K prophylaxis. Report of scientific and standardization subcommitte on perinatal haemostasis. Thrombos Haemost.

[21] Isarangkura PB, Pintadit P, Tejavej A, Siripoonya P, Chulajata C, Gren GM (1986). Vitamin K prophylaxis in the neonate by oral route and its significanse in reducing infant mortality and morbidity. J Med Assoc Thai.

[22] Motohara K, Matsukura M, Matsuda I, Iribe K, Ikeda T, Kondo Y, Yonekubo A, Yamamoto Y, Tsuchiya F (1984). Severe vitamin K deficiency in breast-fed infants. J Pediatr.

[23] Shearer MJ (1995). Vitamin K. Lancet.

[24] Pereira SP, Shearer MJ, Williams R, Mieli-Vergani G (2003). Intestinal absorption of mixed micellar phylloquinone (vitamin K1) is unreliable in infants with conjugated hyperbilirubinaemia: implications for oral prophylaxis of vitamin K deficiency bleeding. Arch Dis Child Fetal Neonatal Ed.

[25] Sato H, Node Y, Araki T, Ohhaski K, Harada N, Yamamoto Y (2000). Intracranial hemorrhage due to vitamin K deficiency associated with congenital biliary atresia. A case report. Neurosurg Emerg.

[26] Ertekin V, Selimoğlu MA, Gursan N, Ozturk CF (2005). Image and diagnosis. Intracranial haemorrhage due to vitamin K deficiency, as the first symptom of extrahepatic biliary atresia. West Indian Med J.

[27] Bor O Akgun N, Yakut A, Sarhuş F, Köse S (2000) Late hemorrhagic disease of the newborn. Pediatr Int 42:64–66 10.1046/j.1442-200x.2000.01173.x 10703238

[28] Pooni PA, Singh D, Singh H, Jain BK (2003). Intracranial hemorrhage in late hemorrhagic disease of the newborn. Indian Pediatr.

[29] von Kries R, Göbel U (1992) Vitamin K prophylaxis and late hemorrhagic disease of the newborn. Acta Pediatr Scand 81:655–657

[30] Committee on Nutrition, American Academy of Pediatrics (1961). Vitamin K compounds and water- soluble analogues: use in therapy and prophylaxis in pediatrics. Pediatrics.

